# Influenza vaccine in chronic obstructive pulmonary disease among elderly male veterans

**DOI:** 10.1371/journal.pone.0262072

**Published:** 2022-01-04

**Authors:** Yinong Young-Xu, Jeremy Smith, Joshua Nealon, Salaheddin M. Mahmud, Robertus Van Aalst, Edward W. Thommes, Nabin Neupane, Jason K. H. Lee, Ayman Chit

**Affiliations:** 1 Clinical Epidemiology Program, Veterans Affairs Medical Center, White River Junction, Vermont, United States of America; 2 Department of Psychiatry, Geisel School of Medicine at Dartmouth, Hanover, New Hampshire, United States of America; 3 Sanofi Pasteur, Medical Evidence Generation, Lyon, France; 4 Department of Community Health Sciences, College of Medicine, University of Manitoba, Winnipeg, Manitoba, Canada; 5 Faculty of Medical Sciences, University of Groningen, Groningen, The Netherlands; 6 Sanofi Pasteur, Swiftwater, Pennslyvania, United States of America; 7 Department of Mathematics & Statistics, University of Guelph, Guelph, Ontario, Canada; 8 Leslie Dan School of Pharmacy, University of Toronto, Toronto, Ontario, Canada; 9 Sanofi Pasteur, Toronto, Ontario, Canada; The Chinese University of Hong Kong, HONG KONG

## Abstract

**Background:**

Prior studies have established those elderly patients with chronic obstructive pulmonary disease (COPD) are at elevated risk for developing influenza-associated complications such as hospitalization, intensive-care admission, and death. This study sought to determine whether influenza vaccination could improve survival among elderly patients with COPD.

**Materials/Methods:**

This study included Veterans (age ≥ 65 years) diagnosed with COPD that received care at the United States Veterans Health Administration (VHA) during four influenza seasons, from 2012–2013 to 2015–2016. We linked VHA electronic medical records and Medicare administrative files to Centers for Disease Control and Prevention National Death Index cause of death records as well as influenza surveillance data. A multivariable time-dependent Cox proportional hazards model was used to compare rates of mortality of recipients of influenza vaccination to those who did not have records of influenza vaccination. We estimated hazard ratios (HRs) adjusted for age, gender, race, socioeconomic status, comorbidities, and healthcare utilization.

**Results:**

Over a span of four influenza seasons, we included 1,856,970 person-seasons of observation where 1,199,275 (65%) had a record of influenza vaccination and 657,695 (35%) did not have a record of influenza vaccination. After adjusting for comorbidities, demographic and socioeconomic characteristics, influenza vaccination was associated with reduced risk of death during the most severe periods of influenza seasons: 75% all-cause (HR = 0.25; 95% CI: 0.24–0.26), 76% respiratory causes (HR = 0.24; 95% CI: 0.21–0.26), and 82% pneumonia/influenza cause (HR = 0.18; 95% CI: 0.13–0.26). A significant part of the effect could be attributed to “healthy vaccinee” bias as reduced risk of mortality was also found during the periods when there was no influenza activity and before patients received vaccination: 30% all-cause (HR = 0.70; 95% CI: 0.65–0.75), 32% respiratory causes (HR = 0.68; 95% CI: 0.60–0.78), and 51% pneumonia/influenza cause (HR = 0.49; 95% CI: 0.31–0.78). However, as a falsification study, we found that influenza vaccination had no impact on hospitalization due to urinary tract infection (HR = 0.97; 95% CI: 0.80–1.18).

**Conclusions:**

Among elderly patients with COPD, influenza vaccination was associated with reduced risk for all-cause and cause-specific mortality.

## Introduction

The burden of illness caused by seasonal influenza varies by age and the presence of preexisting medical conditions [1,2]. Serious medical complications leading to hospitalizations and deaths are greatest among persons aged 65 years and older, and chronic obstructive pulmonary disease (COPD) is the third leading cause of death in this population [3]. Overall, an estimated 15 million US adults have been diagnosed with COPD, with 5 million among those aged 65 or older (hereinafter referred to as elderly) [4].

An influenza infection is a dangerous event for patients with COPD, one that could lead to severe complications and even death, especially among the elderly [5]. Patients with COPD are at increased risk for respiratory failure and often exhibit signs of frailty [6]. Any respiratory infection, including influenza, can cause inflammation and constricted airways, making it difficult to breathe an adequate amount of oxygen. This inflammation can then lead to an increase in COPD exacerbations [7]. For these reasons, individuals with COPD are recommended to receive seasonal influenza vaccination [8].

Very few studies have examined whether influenza vaccination may be beneficial in high-risk COPD patients [9]. Although the United States Veteran Healthcare Administration (VHA) has issued guidelines and policies to encourage annual influenza vaccination in patients with COPD [10], the numbers of deaths attributable to influenza in this population are difficult to estimate directly because influenza infections are typically not confirmed virologically nor specified on hospital discharge forms or death certificates. In addition, many influenza-associated deaths occur from secondary complications when influenza viruses are no longer detectable [11].

In previous studies, influenza vaccination has been associated with a 47% reduction in all-cause mortality during influenza seasons in community-dwelling elderly [12]. As influenza reportedly contributes to at most 10% of the mortality during the influenza seasons, concerns have been raised regarding the validity of observational studies of vaccine effectiveness (VE) and whether these studies adequately addressed confounding and bias [13–16]. Campitelli et al found that influenza vaccination was also associated with a 45% and 26% reduction in all-cause mortality during periods preceding and following influenza seasons, respectively, indicating the presence of residual confounding of comparable magnitude to the estimated benefit of influenza vaccination in their study [17]. All-cause mortality as a study outcome became so questionable that the World Health Organization (WHO) recommended not to include it when evaluating vaccine effectiveness [18]. With limited administrative data at their disposal, Campitelli and colleagues found that “adjustment for functional status indicators, excluding individuals with high one-year predicted mortality at baseline, and moving the start date of follow-up failed to eliminate the refractory confounding” and called instead for “improved data quality, novel data sources, and/or new analytical techniques” in future studies.

Recognizing these challenges, we aimed to investigate whether influenza vaccination among patients with COPD was associated with reduced mortality among a homogenous population of elderly Veterans, while describing potential sources of confounding and bias.

We employed two approaches to address bias, recommended by Jackson and Simonsen [16], among others, that have shown promising results. The first involved analyzing VE during periods of low influenza activity, for example, periods preceding and following influenza seasons. The second was to include a range of outcomes from the general to the specific and compare the strengths of their associated effectiveness, under the overall assumption that effectiveness should rise as outcomes become more specific. We also added a falsification outcome which should not have been affected by influenza vaccination status. In a sensitivity analysis we explored if patients with severe COPD benefited differently from influenza vaccination against mortality. For this we repeated the primary analysis in patients enrolled in VHA Pulmonary Rehabilitation Program.

## Materials and methods

We obtained ethics approval from the institutional review board at White River Junction VA Medical Center (#1037291–7). All study procedures were carried out in compliance with federal and institutional ethical guidelines. The requirement to obtain informed consent from study participants was waived as there was no more than a minimal risk to the privacy of individuals as the data were analyzed anonymously. There was no patient or public involvement in the study.

### Study design and study period

We designed a retrospective, observational cohort study to compare the risk of mortality among influenza vaccine recipients and non-recipients for the 2012/13, 2013/14, 2014/15, and 2015/16 influenza seasons.

### Setting and data sources

As the single largest integrated healthcare system in the US, the VHA of the Department of Veterans Affairs (VA) provides comprehensive health services to nine million enrolled Veterans of the armed forces at 1,255 health care facilities, including 170 medical centers and 1,074 outpatient sites, that can be followed across the entire healthcare continuum [19]. Data for this study were extracted from integrated administrative databases of the VHA in which individual patients were followed longitudinally based on unique identification numbers. Available information electronic medical record (EMR) data include diagnoses, procedures and surgeries, inpatient and outpatient pharmacy utilization, laboratory results, vital signs, and healthcare-related survey responses as structured data elements, in addition to unstructured clinical notes text.

Cause of death data were obtained from death certificates through the National Death Index (NDI), for Veterans who are enrolled in VHA, have received care from the VHA since 1992, or compensation from the Veterans Benefit Administration (VBA) since 2002. NDI death certificate data are available through the Center of Excellence for Suicide Prevention Joint Department of Veterans Affairs and Department of Defense Suicide Data Repository–NDI (extract 22 January 2020) [20]. Finally, Medicare administrative claims data encompassing non-VHA care received for those enrolled in Centers for Medicaid and Medicare Services were also made available through the VA Information Resource Center.

### Study population and influenza vaccination

The study population included all VHA enrollees who turned 65 years or older by July 1^st^ for each of the study seasons, have a COPD diagnosis recorded in at least one healthcare encounter during the year prior, and maintained their enrollment until the end of the season (June 30^th^ for the following year) or until death, whichever occurred earlier. Influenza vaccination was identified using Current Procedural Terminology (CPT) codes (CPT codes: 90655–90659 and Q2034-Q2039; 90662). We required all study subject to have had at least one inpatient or two outpatient encounters in the respiratory season prior to the study period. This restriction improves the probability of capturing comorbidities and reduces the chance that participants were incidental VHA users.

### Influenza season and activity level

**Fig 1:**
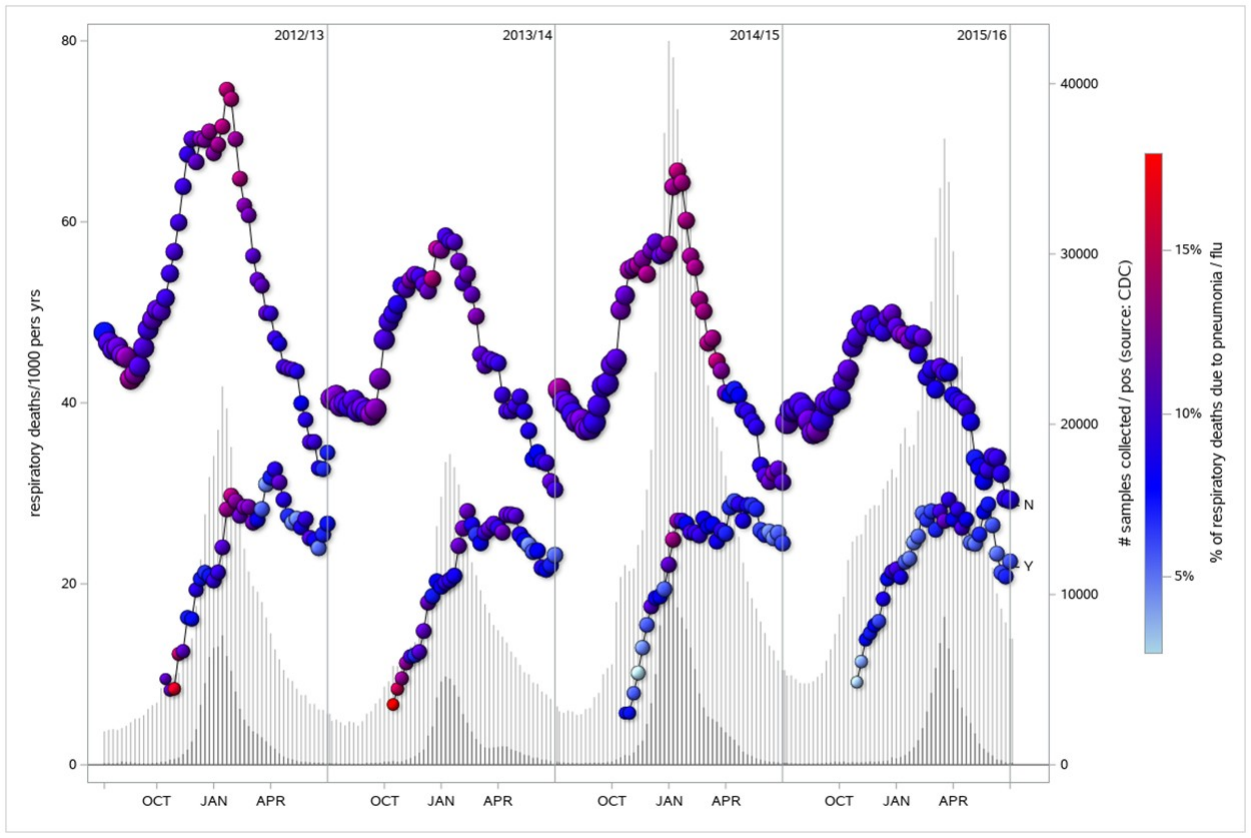
Respiratory mortality in vaccinated vs. non-vaccinated veterans with COPD and corresponding background viral activity, by influenza season, 2012–13 through 2015–16. *Mortality rates are calculated as a 3-week moving average. They are represented on the left-hand y-axis in respiratory-related deaths per 1000 person-years. Bubbles on upper and lower lines are sized according to number of people at risk in each week for non-vaccinated (line ending with ‘N’) and vaccinated (line ending with ‘Y’), respectively. Bubble color ranges from light blue (<5%) to red (>15%) and reflects the percent of respiratory deaths for the week attributable specifically to pneumonia or influenza. Mortality rates for the vaccinated population are unstable during the early season due to small counts of vaccinated individuals; therefore, rates are suppressed until 20% of the overall population is vaccinated in each season. Vertical ‘needles’ are scaled according to the right-hand y-axis and show total weekly influenza samples collected in the United States by the Centers for Disease Control and Prevention (light gray) and total number of those which tested positive (dark gray).

Each influenza season is defined according to the CDC guideline where it begins on July 1^st^ and ends on June 30^th^ of the following year. Each season is then divided into individual weeks where influenza vaccination (lines ending with ‘Y’), influenza viral activity intensity (vertical ‘needles’), and outcomes (left-hand y-axis) were recorded as time-dependent variables (Fig 1).

For each CDC multi-state reporting region [21] we used weekly reports of the percentage of positive influenza tests among all influenza tests performed and total number of tested in each region and categorized influenza activity into five levels according to intensity (Table 1) [22]. Level zero included those weeks with a 0–0.99% positive test rate (on average 3 weeks of the year); other weeks were categorized into four level, with rising degree of influenza viral activity: level 1 (1–3% positivity) included 20 weeks, level 2 (3.1–11% positivity) included approximately 12 weeks, level 3 (11.1–22% positivity) included 11 weeks, and finally, level 4 (22.1–33% positivity) that included about 6 weeks out of each season.

**Table 1:** Observed mortality rates by season and cause of death.

Season/Level		Person-weeks	All-cause	Respiratory	Pneumonia/Influenza		Urinary Tract Infection	CDC Influenza Tests
			Mortality N (Rate, per 100,000)				Hospitalization N (rate)	N (% positive)
2012/13	0 (4)	1,137,318	4,195 (369)	976 (86)	93 (8)	1,133,945	167 (15)	5,826 (0%)
	1 (19)	6,526,467	22,533 (345)	5,405 (83)	527 (8)	6,517,162	592 (9)	71,674 (4%)
	2 (9)	2,976,472	12,091 (406)	2,781 (93)	340 (11)	2,968,927	302 (10)	56,070 (12%)
	3 (12)	3,947,443	16,870 (427)	3,661 (93)	401 (10)	3,936,886	416 (11)	146,588 (24%)
	4 (6)	1,196,741	5,244 (438)	1,126 (94)	106 (9)	1,193,081	143 (12)	107,433 (33%)
2013/14	0 (4)	1,202,356	4,001 (333)	919 (76)	98 (8)	1,198,566	141 (12)	5,876 (0%)
	1 (19)	6,963,105	22,652 (325)	5,138 (74)	508 (7)	6,951,717	642 (9)	81,565 (4%)
	2 (15)	5,743,090	20,298 (353)	4,521 (79)	463 (8)	5,730,589	571 (10)	112,804 (11%)
	3 (10)	3,639,612	14,057 (386)	2,838 (78)	311 (9)	3,630,211	351 (10)	137,295 (22%)
	4 (5)	492,062	1,695 (344)	399 (81)	39 (8)	490,541	62 (13)	44,922 (30%)
2014/15	0	995,867	3,457 (347)	748 (75)	81 (8)	992,552	146 (15)	2,771 (0%)
	1	6,764,028	21,002 (311)	4,900 (72)	449 (7)	6,756,039	550 (8)	130,003 (2%)
	2	3,991,490	14,371 (360)	3,347 (84)	397 (10)	3,982,057	383 (10)	132,931 (7%)
	3	4,128,880	15,901 (385)	3,499 (85)	389 (9)	4,118,929	377 (9)	239,764 (14%)
	4	2,590,498	10,119 (391)	2,138 (83)	243 (9)	2,583,826	242 (9)	288,482 (28%)
2015/16	0	1,240,962	4,067 (328)	917 (74)	87 (7)	1,237,585	132 (11)	9,583 (0%)
	1	9,782,878	32,167 (329)	7,188 (73)	679 (7)	9,765,013	901 (9)	280,686 (1%)
	2	3,712,504	13,042 (351)	2,841 (77)	263 (7)	3,705,164	299 (8)	155,219 (6%)
	3	3,761,239	13,405 (356)	2,985 (79)	288 (8)	3,754,257	297 (8)	246,706 (15%)
	4	832,251	2,895 (348)	650 (78)	64 (8)	830,719	57 (7)	101,813 (22%)
Four Seasons Average								
0		1,144,126	3,930 (343)	890 (78)	90 (8)	1,140,662	147 (13)	6,014 (0%)
1		7,509,120	24,589 (327)	5,658 (75)	541 (7)	7,497,483	671 (9)	140,982 (2%)
2		4,105,889	14,951 (364)	3,373 (82)	366 (9)	4,096,684	389 (9)	114,256 (8%)
3		3,869,294	15,058 (389)	3,246 (84)	347 (9)	3,860,071	360 (9)	192,588 (18%)
4		1,277,888	4,988 (390)	1,078 (84)	113 (9)	1,274,542	126 (10)	135,663 (28%)

### Baseline characteristics and comorbidities

Baseline characteristics were observed in the prior season (e.g., 2012/13 for 2013/14 season). Characteristics measured during the baseline period included demographics, comorbidities, laboratory values, medication, and health care utilization. Demographics included age, sex, race, US geographic region, service-connected disability, and priority level of VHA care. Priority level of VHA care serves as a surrogate measure for socioeconomic status because it is partially based on income and on the capacity for gainful employment [23] Significant comorbidities were defined according to an adaptation of Deyo-Charlson comorbidity score [24]. The comorbidities of interest were chronic health conditions such as cancer, cardiovascular disease, and respiratory disease that could put individuals at elevated risk for complications owing to influenza, as identified by Mullooly and colleagues [2]. Medical conditions were based on clinical diagnosis codes recorded in the VHA electronic medical system during healthcare encounters or in CMS administrative claims data. In addition, we identified patients who underwent pulmonary rehabilitation programs [25] as well as those who needed oxygen therapy. All baseline characteristics were included as covariates in the statistical models.

### Outcomes

Outcomes included mortality caused by cardiovascular (ICD-10, I), respiratory (ICD-10, J), COPD (ICD-10, J), pneumonia (ICD-10, J12-J18), or influenza (ICD-10, J9-J11) disease, as well as all-cause mortality. To compute cause-specific mortality rates, the total number of decedents, with a specified condition coded as a cause of death in the NDI data files in any given week was divided by the total exposure time at risk (i.e., time under the impact of vaccine) by the exposed (e.g., vaccinated) population in the week 14 days prior.

### Statistical methods

We used standardized mean difference (SMD) as a measure of statistical differences between vaccinated and non-vaccinated patients. SMD was initially created for continuous variables and calculated by dividing the difference in mean outcome between groups by the pooled standard deviation of the two groups. This concept was then extended to discrete variables and proportions. The absolute value of this division is then multiplied by 100, with a value greater than 10 denoting statistical significance [26].

Cox proportional hazards models were used to estimate the relative risk of mortality for vaccinated individuals compared to unvaccinated individuals, separately for each week, but combining the data from each influenza activity period. A time-dependent variable for vaccination status was used in the analysis to account for changes in vaccination status. All individuals were classified as unvaccinated at the start of a study season (July 1st) and switched to vaccinated 14 days following receipt of immunization for those who were not vaccinated during the season, they would contribute to non-exposed time only. Date of death is another time-dependent variable in the model. Finally, influenza activity intensity varies throughout any given season, thus making it the third and final time-dependent (5-level categorical) variable in the model. We associated mortality to influenza activity in the two weeks prior to the date of death because we considered this a reasonable duration of time lag between infection and death, as reported elsewhere [27]. All baseline variables not balanced between vaccinated and unvaccinated, i.e., with a SMD>10, were added to the Cox proportional hazards models. Vaccine effectiveness (VE) was expressed as [(1 –fully adjusted hazard ratio (HR))×100].

All tests were two-tailed, and 0.05 was the level of statistical significance. We performed statistical analyses using SAS 9.4 (SAS Institute Inc. Cary, North Carolina).

## Results

Over a span of four influenza seasons, we included 1,856,970 person-seasons of observation where 1,199,275 (65%) had a record of influenza vaccination (hereinafter referred to as vaccinated) and 657,695 (35%) did not have a record of influenza vaccination (not vaccinated). All baseline characteristics are described in Table 2. During the four seasons: 97% of person-time was contributed by males, 80% of non-Hispanic white origin (Table 2). Mean age was 72 (standard deviation [SD]: 10), this varied from 72.7 (SD, 10.0) in those vaccinated to 70.6 (SD, 10.9) in those unvaccinated. Most (86.7%) of vaccine recipients had been vaccinated in the previous season; and 77.9% of Veterans received their vaccination from the VHA. Overall, in this narrowly defined senior, male, Veterans population with COPD, among measured potential confounders, those with a record of influenza vaccination did not differ considerably from those without a record of influenza vaccination. Characteristics of vaccinated and unvaccinated cohorts are described in total in Table 2. Individual seasons are described in S1 Table.

**Table 2:** Baseline characteristics by vaccination status—Study period total.

	Total	Vaccinated	No Vaccine Records	SMD
Population Size	1,690,585 (100.0)	1,113,416 (65.9)	577,169 (34.1)	
Age	72.0 (10.4)	72.7 (10.0)	70.6 (10.9)	19.2
Male	1,644,347 (97.3)	1,085,227 (97.5)	559,120 (96.9)	3.6
Race				
(missing)	76,518 (4.5)	48,315 (4.3)	28,203 (4.9)	2.6
Asian	4,320 (0.3)	2,763 (0.2)	1,557 (0.3)	0.4
American Indian/Alaskan Native	8,948 (0.5)	5,518 (0.5)	3,430 (0.6)	1.3
Black	155,244 (9.2)	90,327 (8.1)	64,917 (11.2)	10.6
Native Hawaiian/Pac Islanders	10,545 (0.6)	6,857 (0.6)	3,688 (0.6)	0.3
White	1,375,920 (81.4)	921,523 (82.8)	454,397 (78.7)	10.3
(declined)	41,270 (2.4)	26,638 (2.4)	14,632 (2.5)	0.9
(unknown)	17,820 (1.1)	11,475 (1.0)	6,345 (1.1)	0.7
Priority				
1	169,576 (10.0)	114,099 (10.2)	55,477 (9.6)	2.1
2	88,324 (5.2)	59,029 (5.3)	29,295 (5.1)	1.0
3	171,887 (10.2)	114,740 (10.3)	57,147 (9.9)	1.3
4	8,828 (0.5)	5,408 (0.5)	3,420 (0.6)	1.5
5	479,782 (28.4)	295,215 (26.5)	184,567 (32.0)	12.0
6	116,990 (6.9)	76,422 (6.9)	40,568 (7.0)	0.6
7	139,058 (8.2)	95,471 (8.6)	43,587 (7.6)	3.8
8	516,140 (30.5)	353,032 (31.7)	163,108 (28.3)	7.5
Disability				
0	62,275 (3.7)	40,060 (3.6)	22,215 (3.8)	1.3
>0–30	158,109 (9.4)	105,384 (9.5)	52,725 (9.1)	1.1
31–70	149,436 (8.8)	100,696 (9.0)	48,740 (8.4)	2.1
>70	106,209 (6.3)	70,903 (6.4)	35,306 (6.1)	1.0
(missing)	1,214,556 (71.8)	796,373 (71.5)	418,183 (72.5)	2.1
Rurality				
(missing)	1,548 (0.1)	0,773 (0.1)	0,775 (0.1)	2.0
Highly Rural	28,590 (1.7)	18,356 (1.6)	10,234 (1.8)	1.0
Rural	666,870 (39.4)	443,184 (39.8)	223,686 (38.8)	2.1
Urban	993,577 (58.8)	651,103 (58.5)	342,474 (59.3)	1.7
Vaccination Prior Season	1,207,390 (71.4)	965,777 (86.7)	241,613 (41.9)	106.0
Vaccination Record Source				
CMS		246,353 (22.1)		
VA		867,063 (77.9)		
Influenza Vaccination type				
High Dose Trivalent		120,251 (10.8)		
Standard Quadrivalent		20,657 (1.9)		
Standard Trivalent		972,508 (87.3)		
Pneumovax	270,335 (16.0)	190,267 (17.1)	80,068 (13.9)	8.9
A1C				
(missing)	739,643 (43.8)	472,918 (42.5)	266,725 (46.2)	7.5
High	514,784 (30.5)	355,865 (32.0)	158,919 (27.5)	9.7
Low	3,951 (0.2)	2,419 (0.2)	1,532 (0.3)	1.0
Normal	432,207 (25.6)	282,214 (25.3)	149,993 (26.0)	1.5
Renal Function Test				
(missing)	586,965 (34.7)	382,802 (34.4)	204,163 (35.4)	2.1
High	159,950 (9.5)	103,454 (9.3)	56,496 (9.8)	1.7
Low	292,373 (17.3)	200,819 (18.0)	91,554 (15.9)	5.8
Normal	651,297 (38.5)	426,341 (38.3)	224,956 (39.0)	1.4
Total Cholesterol				
(missing)	340,288 (20.1)	205,338 (18.4)	134,950 (23.4)	12.2
High	209,311 (12.4)	130,781 (11.7)	78,530 (13.6)	5.6
Low	160,304 (9.5)	108,829 (9.8)	51,475 (8.9)	2.9
Normal	980,682 (58.0)	668,468 (60.0)	312,214 (54.1)	12.0
COPD Severity				
Exacerbation Hospitalization	39,490 (2.3)	25,358 (2.3)	14,132 (2.4)	1.1
Pulmonary Rehab	274,461 (16.2)	188,675 (16.9)	85,786 (14.9)	5.7
Oxygen Therapy	35,043 (2.1)	23,869 (2.1)	11,174 (1.9)	1.5
Medical Conditions				
Congestive Heart Failure	357,276 (21.1)	236,414 (21.2)	120,862 (20.9)	0.7
Cancer	260,737 (15.4)	176,213 (15.8)	84,524 (14.6)	3.3
Metastasis Cancer	23,275 (1.4)	12,681 (1.1)	10,594 (1.8)	5.8
Cardiovascular Disease	190,971 (11.3)	125,659 (11.3)	65,312 (11.3)	0.1
Dementia	47,689 (2.8)	26,920 (2.4)	20,769 (3.6)	6.9
Diabetes with Complication	170,896 (10.1)	120,468 (10.8)	50,428 (8.7)	7.0
Diabetes without Complication	568,885 (33.7)	391,797 (35.2)	177,088 (30.7)	9.6
Human Immunodeficiency Virus	6,143 (0.4)	4,386 (0.4)	1,757 (0.3)	1.5
Hypertension with Complication	220,465 (13.0)	144,622 (13.0)	75,843 (13.1)	0.4
Hypertension without Complication	1,145,603 (67.8)	774,955 (69.6)	370,648 (64.2)	11.5
Liver Disease—Mild	75,130 (4.4)	45,695 (4.1)	29,435 (5.1)	4.8
Liver Disease–Severe	9,876 (0.6)	5,848 (0.5)	4,028 (0.7)	2.2
Myocardial Infarction History	145,308 (8.6)	92,757 (8.3)	52,551 (9.1)	2.7
Paraplegia/Hemiplegia	20,468 (1.2)	12,559 (1.1)	7,909 (1.4)	2.2
Peptic Ulcer Disease	26,736 (1.6)	17,001 (1.5)	9,735 (1.7)	1.3
Peripheral Vascular Disease	287,880 (17.0)	191,634 (17.2)	96,246 (16.7)	1.4
Rheumatic Arthritis	42,295 (2.5)	29,580 (2.7)	12,715 (2.2)	2.9
Renal Disease	278,405 (16.5)	185,563 (16.7)	92,842 (16.1)	1.6
Renal Failure	278,801 (16.5)	185,820 (16.7)	92,981 (16.1)	1.6
Medication Usage				
Aldosterone	6,450 (0.4)	4,267 (0.4)	2,183 (0.4)	0.1
Antibiotic	829,113 (49.0)	542,128 (48.7)	286,985 (49.7)	2.1
Anticholinergic	606,127 (35.9)	409,513 (36.8)	196,614 (34.1)	5.7
Antidepressant	643,396 (38.1)	425,698 (38.2)	217,698 (37.7)	1.1
Antiepileptic	401,879 (23.8)	266,532 (23.9)	135,347 (23.5)	1.1
Antipsychotic	151,017 (8.9)	94,041 (8.4)	56,976 (9.9)	4.9
Antithrombotic	565,729 (33.5)	374,518 (33.6)	191,211 (33.1)	1.1
Aspirin	430,386 (25.5)	285,871 (25.7)	144,515 (25.0)	1.5
Beta-2 Agonist	949,522 (56.2)	632,581 (56.8)	316,941 (54.9)	3.8
Beta Blocker	762,441 (45.1)	512,137 (46.0)	250,304 (43.4)	5.3
Bronchodilator	1,208,804 (71.5)	808,739 (72.6)	400,065 (69.3)	7.3
Calcium Channel Blocker	490,398 (29.0)	329,788 (29.6)	160,610 (27.8)	4.0
Clopidogrel	164,255 (9.7)	111,939 (10.1)	52,316 (9.1)	3.4
Digoxin	81,627 (4.8)	54,996 (4.9)	26,631 (4.6)	1.5
Diuretics	687,568 (40.7)	462,696 (41.6)	224,872 (39.0)	5.3
Methylxanthines	31,340 (1.9)	22,649 (2.0)	8,691 (1.5)	4.0
Mucolytics	12,415 (0.7)	8,287 (0.7)	4,128 (0.7)	0.3
Opiate	775,803 (45.9)	504,523 (45.3)	271,280 (47.0)	3.4
Protein pump inhibitor	809,344 (47.9)	549,956 (49.4)	259,388 (44.9)	8.9
Renin Angio Inhibitor	751,557 (44.5)	508,227 (45.6)	243,330 (42.2)	7.0
Spironolactone	69,795 (4.1)	46,960 (4.2)	22,835 (4.0)	1.3
Statin	1,058,124 (62.6)	729,215 (65.5)	328,909 (57.0)	17.5
Steroid	480,153 (28.4)	315,036 (28.3)	165,117 (28.6)	0.7

**Note.** Vaccinated and unvaccinated groups are well balanced in most of the variables. Where there are significant differences, variables were included in the final, parsimonious multivariable model.

Averaging the 4 seasons, the crude, observed all-cause mortality per 100,000 person-weeks increased from 344 to 380 when viral activity increased from level 0 to 4. Similarly, we observed a trend between higher respiratory (from 78 to 84 per 100,000) and influenza and pneumonia (from 7.9 to 8.5 per 100,000) with increasing influenza activity levels from 0 to 4.

With multivariable Cox proportional hazard models, adjusting for measured confounding factors listed in Table 2, we identified associations between influenza vaccination status and mortality from influenza and pneumonia, respiratory causes, and all cases in inverse relation with rising level of influenza activity (Table 3). Over the four seasons, the hazard ratio for all-cause mortality decreased from 0.70 to 0.25 when viral activity increased from level 0 to 4, suggesting an increased benefit of vaccination in periods with higher viral activity. The same trend was observed in respiratory mortality (hazard ratios decreased from 0.68 to 0.24) and influenza and pneumonia mortality (0.49 to 0.18). For all three outcomes, statistically significant negative associations were observed even when no influenza was circulating (level zero), indicative of residual confounding. These inverse associations between hazard ratios and viral activity levels were also observed in respiratory mortality among COPD patients who enrolled in pulmonary rehabilitation programs as our sensitivity analysis demonstrated—hazard ratios steadily decreased from 0.61 to 0.23 when viral activity increased from level 0 to 4. In contrast, no significant associations were observed between influenza vaccination status and our falsification outcome, UTI hospitalization, irrespective of circulating influenza activity, where the associations hovered around the null (hazard ratios were 0.99, 0.97, and 0.97 during the three periods with the highest influenza viral activity). There were still indications of health vaccinee bias during the 2012/13 and 2013/14 season where, like mortality rates, a protective effect was observed when no influenza was circulating (level zero).

**Table 3:** Adjusted hazard ratio of influenza vaccination and mortality by influenza activity level.

Outcome	Influenza Burden	2012/13	2013/14	2014/15	2015/16	Overall
All-Cause Mortality	0	0.69 (0.62, 0.78)	0.72 (0.63, 0.81)	0.69 (0.59, 0.82)	0.69 (0.61, 0.78)	**0.70 (0.65, 0.75)**
	1	0.50 (0.48, 0.52)	0.60 (0.58, 0.62)	0.61 (0.59, 0.63)	0.63 (0.60, 0.65)	**0.59 (0.58, 0.60)**
	2	0.36 (0.34, 0.38)	0.42 (0.40, 0.43)	0.50 (0.48, 0.53)	0.44 (0.42, 0.46)	**0.43 (0.42, 0.44)**
	3	0.26 (0.25, 0.27)	0.27 (0.26, 0.29)	0.34 (0.33, 0.36)	0.41 (0.39, 0.43)	**0.32 (0.31, 0.32)**
	4	0.21 (0.19, 0.23)	0.24 (0.21, 0.29)	0.23 (0.22, 0.25)	0.41 (0.37, 0.45)	**0.25 (0.24, 0.26)**
Respiratory Mortality	0	0.72 (0.58, 0.89)	0.64 (0.50, 0.82)	0.77 (0.54, 1.08)	0.63 (0.49, 0.81)	**0.68 (0.60, 0.78)**
	1	0.53 (0.49, 0.56)	0.62 (0.58, 0.67)	0.64 (0.59, 0.68)	0.66 (0.62, 0.71)	**0.61 (0.59, 0.64)**
	2	0.36 (0.32, 0.40)	0.43 (0.39, 0.46)	0.50 (0.45, 0.55)	0.51 (0.46, 0.57)	**0.45 (0.43, 0.47)**
	3	0.29 (0.26, 0.32)	0.28 (0.25, 0.31)	0.36 (0.33, 0.40)	0.43 (0.39, 0.47)	**0.33 (0.32, 0.35)**
	4	0.20 (0.17, 0.25)	0.19 (0.13, 0.27)	0.22 (0.18, 0.25)	0.46 (0.38, 0.56)	**0.24 (0.21, 0.26)**
Pneumonia/Influenza Mortality	0	0.46 (0.21, 1.04)	0.86 (0.39, 1.91)	0.27 (0.08, 0.97)	0.48 (0.20, 1.12)	**0.49 (0.31, 0.78)**
	1	0.52 (0.41, 0.66)	0.69 (0.54, 0.89)	0.51 (0.40, 0.65)	0.61 (0.47, 0.78)	**0.58 (0.51, 0.66)**
	2	0.32 (0.23, 0.45)	0.36 (0.27, 0.47)	0.47 (0.35, 0.64)	0.40 (0.27, 0.58)	**0.38 (0.33, 0.45)**
	3	0.29 (0.21, 0.40)	0.24 (0.17, 0.34)	0.25 (0.18, 0.35)	0.30 (0.21, 0.43)	**0.27 (0.23, 0.32)**
	4	0.21 (0.10, 0.41)	0.36 (0.13, 0.99)	0.12 (0.07, 0.21)	0.40 (0.21, 0.78)	**0.18 (0.13, 0.26)**
Sens: Pulmonary Rehabilitation	0	0.64 (0.39, 1.05)	0.69 (0.41, 1.16)	0.76 (0.35, 1.66)	0.43 (0.25, 0.73)	**0.61 (0.45, 0.81)**
(Respiratory Mortality)	1	0.46 (0.39, 0.53)	0.66 (0.57, 0.78)	0.58 (0.50, 0.67)	0.59 (0.51, 0.69)	**0.57 (0.53, 0.62)**
	2	0.37 (0.30, 0.47)	0.39 (0.33, 0.46)	0.47 (0.38, 0.59)	0.49 (0.39, 0.60)	**0.43 (0.38, 0.47)**
	3	0.21 (0.17, 0.26)	0.30 (0.24, 0.38)	0.30 (0.24, 0.37)	0.37 (0.31, 0.46)	**0.29 (0.26, 0.32)**
	4	0.12 (0.07, 0.20)	0.15 (0.07, 0.34)	0.25 (0.18, 0.34)	0.59 (0.39, 0.89)	**0.23 (0.18, 0.29)**
Sens: Falsification Outcome (UTI)	0	0.75 (0.35, 1.59)	0.61 (0.29, 1.29)	1.66 (0.67, 4.10)	3.87 (1.20, 12.41)	**1.32 (0.83, 2.09)**
	1	1.13 (0.89, 1.43)	1.22 (0.96, 1.55)	1.06 (0.85, 1.32)	1.18 (0.97, 1.42)	**1.15 (1.03, 1.28)**
	2	0.86 (0.60, 1.23)	0.97 (0.79, 1.20)	1.14 (0.85, 1.54)	0.98 (0.76, 1.27)	**0.99 (0.87, 1.13)**
	3	1.17 (0.93, 1.47)	0.81 (0.64, 1.01)	0.97 (0.75, 1.26)	0.93 (0.74, 1.19)	**0.97 (0.86, 1.09)**
	4	0.87 (0.61, 1.24)	0.49 (0.28, 0.85)	1.10 (0.83, 1.45)	1.14 (0.65, 2.02)	**0.97 (0.80, 1.18)**

## Discussion

In our analysis of four influenza seasons from 2012/13 to 2015/16, we identified strong associations, after controlling for multiple confounders, between influenza vaccination and mortality from a variety of causes in a predominantly white, male, senior population with COPD. These associations were strongest when influenza activity was at its highest but persisted even when influenza viruses were hardly circulating (level 0), which, discounting time lags between infection and death, provided strong evidence for residual confounding and indicated that vaccine recipients were at lower risk of death irrespective of the protective benefits of vaccination.

To address this issue of confounding and try to define a range of plausible associations we adopted the same approach used by Campitelli et al, whereby associations when influenza activity is exceptionally low were used as a measure of residual confounding. We expanded on this by creating an ordinal variable based on influenza viral positivity rates to measure variations in hazard ratio as a function of viral activity. Campitelli and colleagues defined influenza seasons as the first and last occurrences of two consecutive weeks with at least 5% of influenza isolates testing, corresponding to our level 3 and level 4 periods (Fig 1). Their study population included 25,922 Ontario residents over age 65 who responded to population health surveys. In comparison, our study population included close to half a million patients each season.

**Fig 2:**
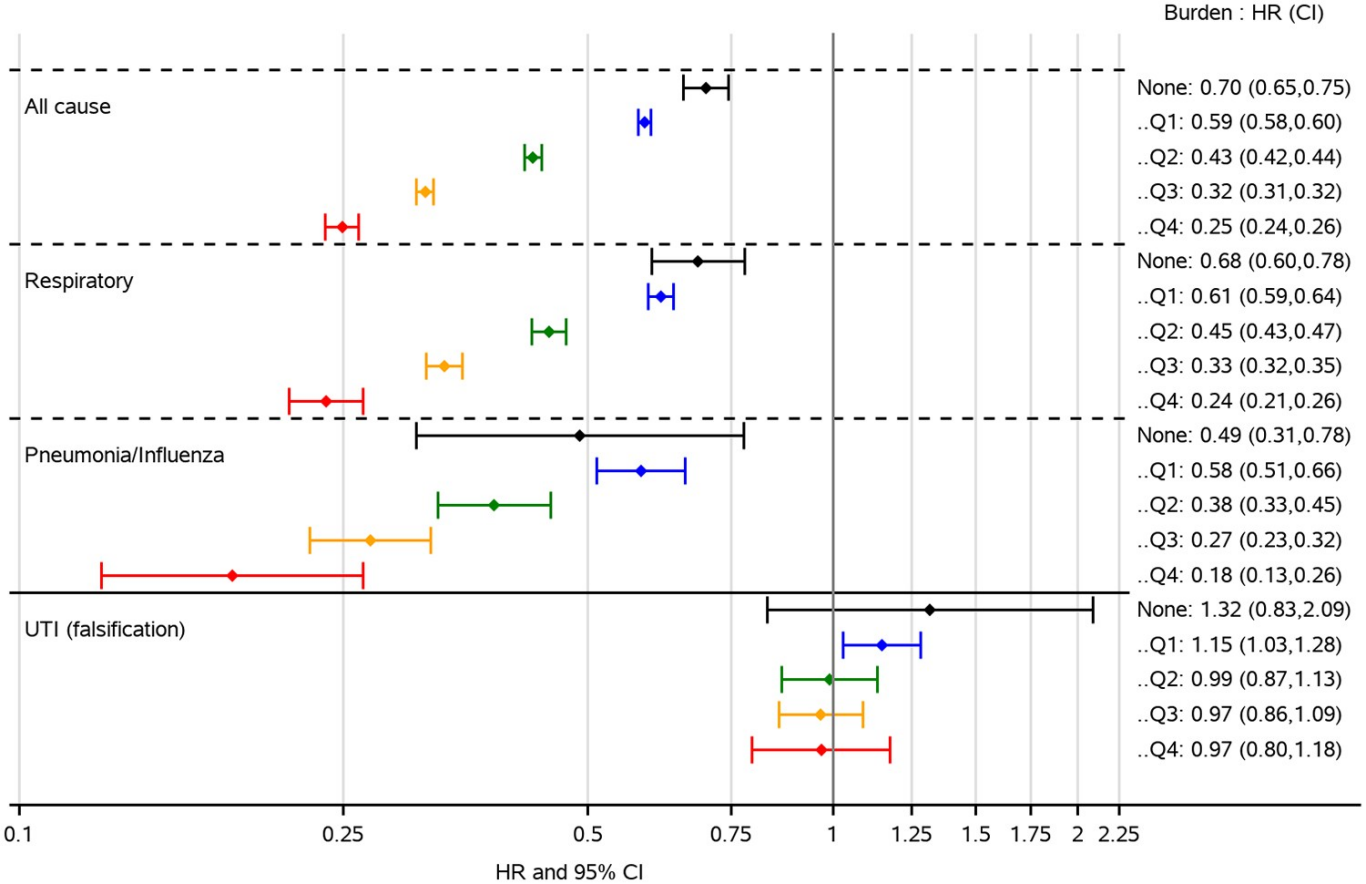
Vaccine effectiveness by cause of death and influenza viral activity level. *UTI—urinary tract infection hospitalization.

We hypothesized that, as the levels of influenza activity increase from low to high; and as the specificity of outcomes in our analysis increased, hazard ratios would decrease. This was borne out by our analysis: hazard ratios for all-cause mortality declined from 0.59 when influenza circulation was lowest (level 1), to 0.25 at the peak of the season during which the average positive influenza test rate was 30% (level 4). We expected associations would be strongest for mortality from more specific respiratory causes because influenza vaccine was the exposure of interest and the underlying COPD in the study population and indeed observed hazard ratios of 0.24 and 0.18, respectively (Fig 2).

The true protective effect of influenza vaccine appears to be dependent on influenza activity level and on an accurate estimate of the healthy vaccinee bias in the specific study population. If we were to assume that the VE at level 3, about 20% positive test rate, was still reflecting some residual confounding, and were to take the difference between VE at level 3 and VE at level 4, we would have arrived at an estimation of VE against all-cause mortality at 7%, which appears to be plausible since influenza has been estimated to account for about 10% of excess deaths during influenza seasons among elderly Americans [28].

Using the same approach, but with an outcome with specificity–respiratory deaths, we took the difference of VE at level 2, about 15% positive test rate, and VE at level 4 and found a VE of 21%. If we still used level 3 VE as a measure of residual confounding, we would have found a VE of 9% against respiratory mortality. Repeating this approach once more, we found a VE of 20% against influenza/pneumonia death, using level 2 as a reference; and a VE of 11%, using level 3 as a reference. In summary, using VEs found at level 3 influenza activity, about 20% positive test rate, as a measure of residual confounding, we would have found VEs to be 7%, 9%, and 11% against all-cause, respiratory, and influenza/pneumonia deaths during periods with the highest level of influenza activity, respectively. And evidence suggests that the latter two could be 20% or higher had we compared to VEs from a lower level of influenza activity. In other words, seasonal influenza vaccination could have a substantial impact as nearly 3,800 VHA patients die annually from respiratory and circulatory complications associated with seasonal influenza infections [29].

Campitelli et al included all-cause mortality as an outcome and identified reductions in vaccine recipients of 45% and 26%, during pre and post influenza periods, respectively, effects which were comparable to the 39% reduction observed while influenza was circulating. We also included this outcome and observed reductions of similar magnitude: an average of 42% reduction at levels 0, 1, 2 when <11% of respiratory samples were testing positive for influenza.

Our findings underling the WHO’s guideline that “it is very difficult to accurately estimate VE in view of…healthy vaccinee bias” which strongly impacts the non-specific endpoint like all-cause mortality [18]. Indeed, despite controlling for potential confounders, strong protective associations persisted against the multiple endpoints included in our study even when influenza was not circulating. Similar trends were also observed using relatively specific endpoints of respiratory/influenza and pneumonia mortality. Our findings that associations strengthened as influenza activity increased is a persuasive indicator of the protective benefit of vaccination and while it may be possible to quantify vaccine effectiveness after adjusting for measured residual confounding, formal quantification of that benefit was not an objective of our study.

### Strengths

Our study was conducted in a large, highly comorbid population in which influenza vaccination rate was about 65% during the study period, providing ample study subjects and events to evaluate the impact of seasonal influenza vaccination on mortality outcomes. Baseline demographics of vaccinated and unvaccinated veterans appeared broadly similar, and most vaccination took place within the VA, a reassuring indicator that we are not missing medical visits elsewhere. We carried out a falsification analysis using hospitalization due to UTI, finding associations close to the null, and with no trends with influenza viral activity. We also carried out a sensitivity analysis, focusing on COPD Veterans enrolled in VHA pulmonary rehabilitation programs, and observed the same association between increased protective effect and rising viral activity levels.

### Limitations

In this study, we had records of vaccinations stored in VA and CMS databases. Hence, if some patients received influenza vaccines from elsewhere—for instance, at a community center—this would not have been recorded. However, if this were the case, these patients would have been classified as unvaccinated, and this would likely only serve to weaken any potential association between influenza vaccination and outcome. Furthermore, we lacked data on important clinical variables such as lung function test results to better assessed the severity of a patient’s underlying COPD. Finally, CDC’s causes-of-death data, though the gold standard, are not without flaws, one of which is a lack of standardization [30] that may pose challenges for comparison. Because influenza and pneumonia are comparatively rare as causes of death, they might be more subjective an outcome, especially outside the peak influenza period, as under-diagnosing could lead to variation in the assignment of underlying cause of death. As a result, we did not always see that VE increased as outcomes became more specific from all-cause to respiratory and then to influenza/pneumonia. And although our data suggest that specificity improves as influenza activity intensifies, we do not have patient level data to confirm an influenza infection nor chart reviews to adjudicate cause of deaths.

We constructed a longitudinal dataset with multiple time periods (in weeks), each with its specific viral activity and repeat measurements. There are a few issues to be considered when analysing repeated measures. One is the correlation between measures from the same patient. One example, a patient’s medication use might be correlated from season to season. Our main analysis was season specific, and only the summary results, arrived through meta-analysis, included all 4 seasons. Within each season, the baseline variables (Table 2) remain constant. Only influenza vaccination, influenza viral activity intensity, and outcomes were recorded as time-dependent variables. Another, in this case, is to account for any people who do not appear in all periods (censoring, death). This was accomplished by our survival model. Finally, influenza does not impact mortality right away; consequently, we added a two-week lag time between the viral activity periods and observed mortality.

## Conclusion

Our effort at improving specificity regarding study population (COPD), influenza activity (5 levels), and outcomes (3) resulted in a more detailed examination of influenza vaccine effectiveness against mortality in this vulnerable population, but we were not able to eliminate residual confounding. In the end, we presented a range of VEs by levels of specificity, instead of delivering a single “take-home” vaccine effectiveness estimate at the risk of being simplistic. These more nuanced findings perhaps could provide healthcare professionals and the public greater confidence that influenza vaccines are protective, after carefully accounting for the healthy vaccinee bias.

## Supporting information

S1 Fig Patient selection. (TIF)

S2 Fig Levels of viral activity over a season. (TIF)

S1 Table Characteristics of vaccinated and unvaccinated cohorts by individual seasons. (PDF)
