# Supplementary figures and images for: Influenza vaccine in chronic obstructive pulmonary disease among elderly male veterans
Source: PLoS One. 2022 Jan 4;17(1):e0262072. doi: 10.1371/journal.pone.0262072 (PMC8726500; doi:10.1371/journal.pone.0262072)

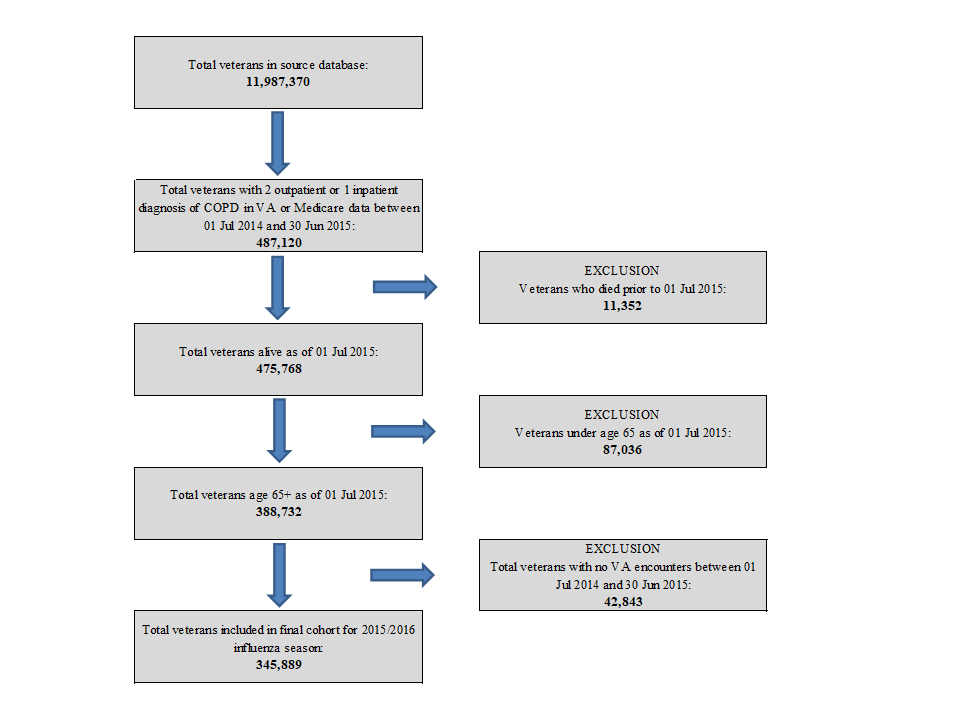

Supplement: S1 Fig — (TIF) [file pone.0262072.s001.tif]

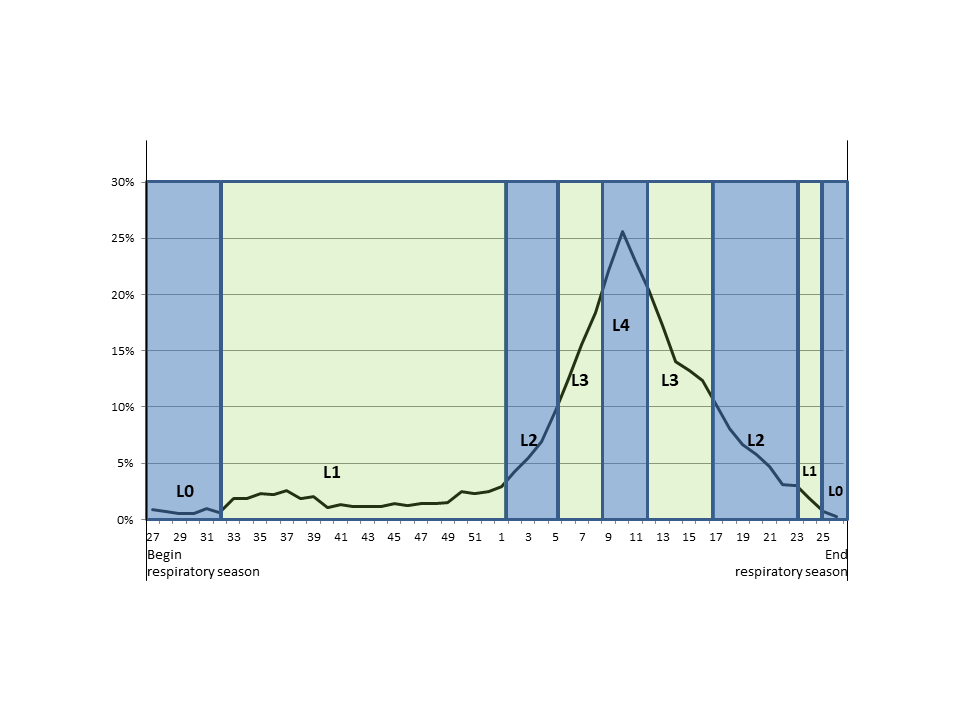

Supplement: S2 Fig — (TIF) [file pone.0262072.s002.tif]
