# Supplementary material for: Influenza vaccine in chronic obstructive pulmonary disease among elderly male veterans
Source: PLoS One. 2022 Jan 4;17(1):e0262072. doi: 10.1371/journal.pone.0262072 (PMC8726500; doi:10.1371/journal.pone.0262072)
Supplement: S1 Table — (PDF) [file pone.0262072.s003.pdf]

	2012/13				2013/14				2014/15				2015/16			
	Total	Vaccinated	Unvaccinated	SMD	Total	Vaccinated	Unvaccinated	SMD	Total	Vaccinated	Unvaccinated	SMD	Total	Vaccinated	Unvaccinated	SMD
Population	420,207 (100.0)	290,985 (69.2)	129,222 (30.8)	83.4	421,099 (100.0)	289,445 (68.7)	131,654 (31.3)	80.8	416,667 (100.0)	277,117 (66.5)	139,550 (33.5)	70.0	432,612 (100.0)	255,869 (59.1)	176,743 (40.9)	37.2
Age	71.8 (10.6)	72.5 (10.2)	70.5 (11.1)	18.6	71.9 (10.5)	72.5 (10.2)	70.7 (11.0)	17.5	72.0 (10.3)	72.7 (10.0)	70.7 (10.8)	18.9	72.1 (10.2)	73.0 (9.8)	70.7 (10.6)	22.8
Male	409,463 (97.4)	283,970 (97.6)	125,493 (97.1)	3.0	409,976 (97.4)	282,302 (97.5)	127,674 (97.0)	3.4	405,020 (97.2)	269,834 (97.4)	135,186 (96.9)	3.0	419,888 (97.1)	249,121 (97.4)	170,767 (96.6)	4.4
Race																
(missing)	25,245 (6.0)	16,638 (5.7)	8,607 (6.7)	3.9	20,777 (4.9)	13,398 (4.6)	7,379 (5.6)	4.4	16,675 (4.0)	10,330 (3.7)	6,345 (4.5)	4.1	13,821 (3.2)	7,949 (3.1)	5,872 (3.3)	1.2
Asian	1,040 (0.2)	0,691 (0.2)	0,349 (0.3)	0.6	1,029 (0.2)	0,716 (0.2)	0,313 (0.2)	0.2	1,072 (0.3)	0,719 (0.3)	0,353 (0.3)	0.1	1,179 (0.3)	0,637 (0.2)	0,542 (0.3)	1.1
American Indian / Alaska Native	2,041 (0.5)	1,342 (0.5)	0,699 (0.5)	1.1	2,127 (0.5)	1,373 (0.5)	0,754 (0.6)	1.4	2,297 (0.6)	1,458 (0.5)	0,839 (0.6)	1.0	2,483 (0.6)	1,345 (0.5)	1,138 (0.6)	1.6
Black	36,478 (8.7)	22,332 (7.7)	14,146 (10.9)	11.3	37,830 (9.0)	23,527 (8.1)	14,303 (10.9)	9.3	39,133 (9.4)	23,490 (8.5)	15,643 (11.2)	9.2	41,803 (9.7)	20,978 (8.2)	20,825 (11.8)	12.0
Native Hawaiian / Pacific Islander	2,608 (0.6)	1,801 (0.6)	0,807 (0.6)	0.1	2,652 (0.6)	1,847 (0.6)	0,805 (0.6)	0.3	2,586 (0.6)	1,717 (0.6)	0,869 (0.6)	0.0	2,699 (0.6)	1,492 (0.6)	1,207 (0.7)	1.3
White	338,002 (80.4)	237,964 (81.8)	100,038 (77.4)	10.8	341,903 (81.2)	238,576 (82.4)	103,327 (78.5)	10.0	340,342 (81.7)	229,893 (83.0)	110,449 (79.1)	9.7	355,673 (82.2)	215,090 (84.1)	140,583 (79.5)	11.7
(declined)	10,199 (2.4)	7,090 (2.4)	3,109 (2.4)	0.2	10,276 (2.4)	6,938 (2.4)	3,338 (2.5)	0.9	10,195 (2.4)	6,661 (2.4)	3,534 (2.5)	0.8	10,600 (2.5)	5,949 (2.3)	4,651 (2.6)	2.0
(unknown)	4,594 (1.1)	3,127 (1.1)	1,467 (1.1)	0.6	4,505 (1.1)	3,070 (1.1)	1,435 (1.1)	0.3	4,367 (1.0)	2,849 (1.0)	1,518 (1.1)	0.6	4,354 (1.0)	2,429 (0.9)	1,925 (1.1)	1.4
Priority																
1	42,997 (10.2)	30,406 (10.4)	12,591 (9.7)	2.3	42,861 (10.2)	30,041 (10.4)	12,820 (9.7)	2.1	41,588 (10.0)	28,208 (10.2)	13,380 (9.6)	2.0	42,130 (9.7)	25,444 (9.9)	16,686 (9.4)	1.7
2	21,885 (5.2)	15,305 (5.3)	6,580 (5.1)	0.8	21,795 (5.2)	15,248 (5.3)	6,547 (5.0)	1.3	21,972 (5.3)	14,880 (5.4)	7,092 (5.1)	1.3	22,672 (5.2)	13,596 (5.3)	9,076 (5.1)	0.8
3	41,416 (9.9)	29,029 (10.0)	12,387 (9.6)	1.3	42,530 (10.1)	29,756 (10.3)	12,774 (9.7)	1.9	42,545 (10.2)	28,645 (10.3)	13,900 (10.0)	1.2	45,396 (10.5)	27,310 (10.7)	18,086 (10.2)	1.4
4	2,483 (0.6)	1,618 (0.6)	0,865 (0.7)	1.5	2,250 (0.5)	1,392 (0.5)	0,858 (0.7)	2.3	2,114 (0.5)	1,290 (0.5)	0,824 (0.6)	1.7	1,981 (0.5)	1,108 (0.4)	0,873 (0.5)	0.9
5	122,582 (29.2)	80,044 (27.5)	42,538 (32.9)	11.8	119,878 (28.5)	77,204 (26.7)	42,674 (32.4)	12.6	117,366 (28.2)	72,791 (26.3)	44,575 (31.9)	12.5	119,956 (27.7)	65,176 (25.5)	54,780 (31.0)	12.3
6	24,281 (5.8)	16,530 (5.7)	7,751 (6.0)	1.4	27,657 (6.6)	18,949 (6.5)	8,708 (6.6)	0.3	30,139 (7.2)	20,123 (7.3)	10,016 (7.2)	0.3	34,913 (8.1)	20,820 (8.1)	14,093 (8.0)	0.6
7	34,819 (8.3)	24,977 (8.6)	9,842 (7.6)	3.5	34,702 (8.2)	24,844 (8.6)	9,858 (7.5)	4.0	34,072 (8.2)	23,616 (8.5)	10,456 (7.5)	3.8	35,465 (8.2)	22,034 (8.6)	13,431 (7.6)	3.7
8	129,744 (30.9)	93,076 (32.0)	36,668 (28.4)	7.9	129,426 (30.7)	92,011 (31.8)	37,415 (28.4)	7.4	126,871 (30.4)	87,564 (31.6)	39,307 (28.2)	7.5	130,099 (30.1)	80,381 (31.4)	49,718 (28.1)	7.2
Disability																
0	15,579 (3.7)	10,509 (3.6)	5,070 (3.9)	1.6	15,600 (3.7)	10,514 (3.6)	5,086 (3.9)	1.2	15,255 (3.7)	9,952 (3.6)	5,303 (3.8)	1.1	15,841 (3.7)	9,085 (3.6)	6,756 (3.8)	1.4
>0-30	38,115 (9.1)	26,693 (9.2)	11,422 (8.8)	1.2	39,103 (9.3)	27,338 (9.4)	11,765 (8.9)	1.8	39,193 (9.4)	26,322 (9.5)	12,871 (9.2)	0.9	41,698 (9.6)	25,031 (9.8)	16,667 (9.4)	1.2
31-70	37,238 (8.9)	26,281 (9.0)	10,957 (8.5)	2.0	37,174 (8.8)	26,183 (9.0)	10,991 (8.3)	2.5	36,868 (8.8)	25,208 (9.1)	11,660 (8.4)	2.6	38,156 (8.8)	23,024 (9.0)	15,132 (8.6)	1.5
>70	27,092 (6.4)	19,033 (6.5)	8,059 (6.2)	1.2	26,877 (6.4)	18,677 (6.5)	8,200 (6.2)	0.9	26,103 (6.3)	17,499 (6.3)	8,604 (6.2)	0.6	26,137 (6.0)	15,694 (6.1)	10,443 (5.9)	0.9
(missing)	302,183 (71.9)	208,469 (71.6)	93,714 (72.5)	2.0	302,345 (71.8)	206,733 (71.4)	95,612 (72.6)	2.7	299,248 (71.8)	198,136 (71.5)	101,112 (72.5)	2.1	310,780 (71.8)	183,035 (71.5)	127,745 (72.3)	1.7
Rurality																
(missing)	442 (0.1)	267 (0.1)	175 (0.1)	1.3	399 (0.1)	226 (0.1)	173 (0.1)	1.6	344 (0.1)	167 (0.1)	177 (0.1)	2.2	363 (0.1)	113 (0.0)	250 (0.1)	3.2
Highly Rural	7,119 (1.7)	4,679 (1.6)	2,440 (1.9)	2.1	7,287 (1.7)	4,827 (1.7)	2,460 (1.9)	1.5	7,001 (1.7)	4,508 (1.6)	2,493 (1.8)	1.2	7,183 (1.7)	4,342 (1.7)	2,841 (1.6)	0.7
Rural	165,815 (39.5)	115,086 (39.6)	50,729 (39.3)	0.6	165,946 (39.4)	114,717 (39.6)	51,229 (38.9)	1.5	164,499 (39.5)	110,320 (39.8)	54,179 (38.8)	2.0	170,610 (39.4)	103,061 (40.3)	67,549 (38.2)	4.2
Urban	246,831 (58.7)	170,953 (58.7)	75,878 (58.7)	0.1	247,467 (58.8)	169,675 (58.6)	77,792 (59.1)	0.9	244,823 (58.8)	162,122 (58.5)	82,701 (59.3)	1.5	254,456 (58.8)	148,353 (58.0)	106,103 (60.0)	4.2

Vaccine Prior Season	303,676 (72.3)	251,835 (86.5)	51,841 (40.1)	109.9	304,337 (72.3)	251,898 (87.0)	52,439 (39.8)	112.4	299,327 (71.8)	242,058 (87.3)	57,269 (41.0)	110.3	300,050 (69.4)	219,986 (86.0)	80,064 (45.3)	94.8
Vaccine source																
(missing)	129,222 (30.8)	0 (0.0)	129,222 (100.0)	0.0	131,654 (31.3)	0 (0.0)	131,654 (100.0)	0.0	139,550 (33.5)	0 (0.0)	139,550 (100.0)	0.0	176,743 (40.9)	0 (0.0)	176,743 (100.0)	0.0
CMS	65,032 (15.5)	65,032 (22.3)	0 (0.0)	75.9	59,027 (14.0)	59,027 (20.4)	0 (0.0)	71.6	60,667 (14.6)	60,667 (21.9)	0 (0.0)	74.9	61,627 (14.2)	61,627 (24.1)	0 (0.0)	79.7
VA	225,953 (53.8)	225,953 (77.7)	0 (0.0)	263.6	230,418 (54.7)	230,418 (79.6)	0 (0.0)	279.4	216,450 (51.9)	216,450 (78.1)	0 (0.0)	267.1	194,242 (44.9)	194,242 (75.9)	0 (0.0)	251.1
Vaccine type																
(missing)	129,222 (30.8)	0 (0.0)	129,222 (100.0)	0.0	131,654 (31.3)	0 (0.0)	131,654 (100.0)	0.0	139,550 (33.5)	0 (0.0)	139,550 (100.0)	0.0	176,743 (40.9)	0 (0.0)	176,743 (100.0)	0.0
High Dose	16,824 (4.0)	16,824 (5.8)	0 (0.0)	35.0	19,459 (4.6)	19,459 (6.7)	0 (0.0)	38.0	32,742 (7.9)	32,742 (11.8)	0 (0.0)	51.8	51,226 (11.8)	51,226 (20.0)	0 (0.0)	70.8
Standard Quadrivalent	1,081 (0.3)	1,081 (0.4)	0 (0.0)	8.6	2,535 (0.6)	2,535 (0.9)	0 (0.0)	13.3	7,114 (1.7)	7,114 (2.6)	0 (0.0)	23.0	9,927 (2.3)	9,927 (3.9)	0 (0.0)	28.4
Standard Trivalent	273,080 (65.0)	273,080 (93.8)	0 (0.0)	552.3	267,451 (63.5)	267,451 (92.4)	0 (0.0)	493.2	237,261 (56.9)	237,261 (85.6)	0 (0.0)	345.0	194,716 (45.0)	194,716 (76.1)	0 (0.0)	252.4
Pneumovax	45,034 (10.7)	32,887 (11.3)	12,147 (9.4)	6.2	48,209 (11.4)	35,348 (12.2)	12,861 (9.8)	7.8	51,222 (12.3)	36,373 (13.1)	14,849 (10.6)	7.7	125,870 (29.1)	85,659 (33.5)	40,211 (22.8)	24.0
A1C																
(missing)	196,604 (46.8)	131,827 (45.3)	64,777 (50.1)	9.7	188,931 (44.9)	125,484 (43.4)	63,447 (48.2)	9.7	178,390 (42.8)	114,379 (41.3)	64,011 (45.9)	9.3	175,718 (40.6)	101,228 (39.6)	74,490 (42.1)	5.3
High	122,508 (29.2)	89,459 (30.7)	33,049 (25.6)	11.5	127,538 (30.3)	92,062 (31.8)	35,476 (26.9)	10.7	130,790 (31.4)	91,363 (33.0)	39,427 (28.3)	10.2	133,948 (31.0)	82,981 (32.4)	50,967 (28.8)	7.8
Low	707 (0.2)	462 (0.2)	245 (0.2)	0.7	847 (0.2)	536 (0.2)	311 (0.2)	1.1	1,023 (0.2)	637 (0.2)	386 (0.3)	0.9	1,374 (0.3)	784 (0.3)	590 (0.3)	0.5
Normal	100,388 (23.9)	69,237 (23.8)	31,151 (24.1)	0.7	103,783 (24.6)	71,363 (24.7)	32,420 (24.6)	0.1	106,464 (25.6)	70,738 (25.5)	35,726 (25.6)	0.2	121,572 (28.1)	70,876 (27.7)	50,696 (28.7)	2.2
Renal Function Test																
(missing)	157,173 (37.4)	106,844 (36.7)	50,329 (38.9)	4.6	145,875 (34.6)	98,619 (34.1)	47,256 (35.9)	3.8	138,931 (33.3)	91,090 (32.9)	47,841 (34.3)	3.0	144,986 (33.5)	86,249 (33.7)	58,737 (33.2)	1.0
High	17,802 (4.2)	11,946 (4.1)	5,856 (4.5)	2.1	38,666 (9.2)	27,127 (9.4)	11,539 (8.8)	2.1	49,382 (11.9)	33,261 (12.0)	16,121 (11.6)	1.4	54,100 (12.5)	31,120 (12.2)	22,980 (13.0)	2.5
Low	73,409 (17.5)	53,064 (18.2)	20,345 (15.7)	6.6	74,192 (17.6)	53,188 (18.4)	21,004 (16.0)	6.4	73,353 (17.6)	50,720 (18.3)	22,633 (16.2)	5.5	71,419 (16.5)	43,847 (17.1)	27,572 (15.6)	4.2
Normal	171,823 (40.9)	119,131 (40.9)	52,692 (40.8)	0.3	162,366 (38.6)	110,511 (38.2)	51,855 (39.4)	2.5	155,001 (37.2)	102,046 (36.8)	52,955 (37.9)	2.3	162,107 (37.5)	94,653 (37.0)	67,454 (38.2)	2.4
Cholesterol																
(missing)	82,864 (19.7)	52,532 (18.1)	30,332 (23.5)	13.4	83,030 (19.7)	52,071 (18.0)	30,959 (23.5)	13.7	83,473 (20.0)	50,889 (18.4)	32,584 (23.3)	12.3	90,921 (21.0)	49,846 (19.5)	41,075 (23.2)	9.2
High	51,952 (12.4)	34,151 (11.7)	17,801 (13.8)	6.1	54,203 (12.9)	35,237 (12.2)	18,966 (14.4)	6.6	51,520 (12.4)	32,342 (11.7)	19,178 (13.7)	6.2	51,636 (11.9)	29,051 (11.4)	22,585 (12.8)	4.4
Low	42,211 (10.0)	30,214 (10.4)	11,997 (9.3)	3.7	39,209 (9.3)	27,779 (9.6)	11,430 (8.7)	3.2	38,184 (9.2)	25,856 (9.3)	12,328 (8.8)	1.7	40,700 (9.4)	24,980 (9.8)	15,720 (8.9)	3.0
Normal	243,180 (57.9)	174,088 (59.8)	69,092 (53.5)	12.9	244,657 (58.1)	174,358 (60.2)	70,299 (53.4)	13.8	243,490 (58.4)	168,030 (60.6)	75,460 (54.1)	13.3	249,355 (57.6)	151,992 (59.4)	97,363 (55.1)	8.7
C-Reactive Protein																
(missing)	404,909 (96.4)	280,441 (96.4)	124,468 (96.3)	0.3	405,493 (96.3)	278,771 (96.3)	126,722 (96.3)	0.3	399,977 (96.0)	266,164 (96.0)	133,813 (95.9)	0.8	414,203 (95.7)	245,242 (95.8)	168,961 (95.6)	1.2
High	7,831 (1.9)	5,254 (1.8)	2,577 (2.0)	1.4	8,282 (2.0)	5,509 (1.9)	2,773 (2.1)	1.4	9,107 (2.2)	5,863 (2.1)	3,244 (2.3)	1.4	9,945 (2.3)	5,747 (2.2)	4,198 (2.4)	0.9
Low	1,585 (0.4)	1,137 (0.4)	0,448 (0.3)	0.7	1,781 (0.4)	1,258 (0.4)	0,523 (0.4)	0.6	1,792 (0.4)	1,233 (0.4)	559 (0.4)	0.7	2,005 (0.5)	1,163 (0.5)	842 (0.5)	0.3
Normal	5,882 (1.4)	4,153 (1.4)	1,729 (1.3)	0.8	5,543 (1.3)	3,907 (1.3)	1,636 (1.2)	0.9	5,791 (1.4)	3,857 (1.4)	1,934 (1.4)	0.1	6,459 (1.5)	3,717 (1.5)	2,742 (1.6)	0.8
Blood PH																
(missing)	416,236 (99.1)	288,227 (99.1)	128,009 (99.1)	0.1	417,151 (99.1)	286,703 (99.1)	130,448 (99.1)	0.3	412,859 (99.1)	274,591 (99.1)	138,268 (99.1)	0.1	428,867 (99.1)	253,894 (99.2)	174,973 (99.0)	2.4

High	635 (0.2)	436 (0.1)	199 (0.2)	0.1	719 (0.2)	493 (0.2)	226 (0.2)	0.0	661 (0.2)	421 (0.2)	240 (0.2)	0.5	688 (0.2)	350 (0.1)	338 (0.2)	1.3
Low	562 (0.1)	393 (0.1)	169 (0.1)	0.1	536 (0.1)	361 (0.1)	175 (0.1)	0.2	490 (0.1)	308 (0.1)	182 (0.1)	0.6	531 (0.1)	276 (0.1)	255 (0.1)	1.0
Normal	2,774 (0.7)	1,929 (0.7)	0,845 (0.7)	0.1	2,693 (0.6)	1,888 (0.7)	0,805 (0.6)	0.5	2,657 (0.6)	1,797 (0.6)	0,860 (0.6)	0.4	2,526 (0.6)	1,349 (0.5)	1,177 (0.7)	1.8
Partial pressure of oxygen																
(missing)	419,410 (99.8)	290,606 (99.9)	128,804 (99.7)	4.1	420,341 (99.8)	288,928 (99.8)	131,413 (99.8)	0.1	415,911 (99.8)	276,660 (99.8)	139,251 (99.8)	1.1	431,807 (99.8)	255,412 (99.8)	176,395 (99.8)	0.4
High	78 (0.0)	45 (0.0)	33 (0.0)	0.7	74 (0.0)	50 (0.0)	24 (0.0)	0.1	76 (0.0)	43 (0.0)	33 (0.0)	0.6	101 (0.0)	55 (0.0)	46 (0.0)	0.3
Low	568 (0.1)	256 (0.1)	312 (0.2)	3.8	532 (0.1)	373 (0.1)	159 (0.1)	0.2	522 (0.1)	329 (0.1)	193 (0.1)	0.5	523 (0.1)	312 (0.1)	211 (0.1)	0.1
Normal	151 (0.0)	78 (0.0)	73 (0.1)	1.5	152 (0.0)	94 (0.0)	58 (0.0)	0.6	158 (0.0)	85 (0.0)	73 (0.1)	1.1	181 (0.0)	90 (0.0)	91 (0.1)	0.8
Partial pressure of Carbon Dioxide																
(missing)	419,410 (99.8)	290,606 (99.9)	128,804 (99.7)	4.1	420,340 (99.8)	288,927 (99.8)	131,413 (99.8)	0.1	415,911 (99.8)	276,660 (99.8)	139,251 (99.8)	1.1	431,807 (99.8)	255,412 (99.8)	176,395 (99.8)	0.4
High	232 (0.1)	123 (0.0)	109 (0.1)	1.7	218 (0.1)	151 (0.1)	67 (0.1)	0.1	210 (0.1)	123 (0.0)	87 (0.1)	0.8	218 (0.1)	128 (0.1)	90 (0.1)	0.0
Low	126 (0.0)	67 (0.0)	59 (0.0)	1.2	127 (0.0)	88 (0.0)	39 (0.0)	0.0	129 (0.0)	76 (0.0)	53 (0.0)	0.6	144 (0.0)	74 (0.0)	70 (0.0)	0.6
Normal	439 (0.1)	189 (0.1)	250 (0.2)	3.6	414 (0.1)	279 (0.1)	135 (0.1)	0.2	417 (0.1)	258 (0.1)	159 (0.1)	0.6	443 (0.1)	255 (0.1)	188 (0.1)	0.2
Bicarbonate																
(missing)	386,684 (92.0)	268,511 (92.3)	118,173 (91.4)	3.0	387,575 (92.0)	267,204 (92.3)	120,371 (91.4)	3.2	384,191 (92.2)	256,339 (92.5)	127,852 (91.6)	3.3	399,513 (92.3)	237,205 (92.7)	162,308 (91.8)	3.3
High	14,354 (3.4)	9,719 (3.3)	4,635 (3.6)	1.3	13,654 (3.2)	9,330 (3.2)	4,324 (3.3)	0.3	13,226 (3.2)	8,685 (3.1)	4,541 (3.3)	0.7	13,239 (3.1)	7,576 (3.0)	5,663 (3.2)	1.4
Low	4,326 (1.0)	2,823 (1.0)	1,503 (1.2)	1.9	4,643 (1.1)	2,965 (1.0)	1,678 (1.3)	2.3	4,339 (1.0)	2,711 (1.0)	1,628 (1.2)	1.8	4,478 (1.0)	2,481 (1.0)	1,997 (1.1)	1.6
Normal	14,843 (3.5)	9,932 (3.4)	4,911 (3.8)	2.1	15,227 (3.6)	9,946 (3.4)	5,281 (4.0)	3.0	14,911 (3.6)	9,382 (3.4)	5,529 (4.0)	3.1	15,382 (3.6)	8,607 (3.4)	6,775 (3.8)	2.5
Oxygen Saturation																
(missing)	411,991 (98.0)	285,378 (98.1)	126,613 (98.0)	0.7	413,206 (98.1)	284,126 (98.2)	129,080 (98.0)	0.9	409,424 (98.3)	272,367 (98.3)	137,057 (98.2)	0.6	424,924 (98.2)	251,234 (98.2)	173,690 (98.3)	0.6
High	769 (0.2)	528 (0.2)	241 (0.2)	0.1	758 (0.2)	537 (0.2)	221 (0.2)	0.4	815 (0.2)	555 (0.2)	260 (0.2)	0.3	867 (0.2)	489 (0.2)	378 (0.2)	0.5
Low	4,232 (1.0)	2,882 (1.0)	1,350 (1.0)	0.5	4,011 (1.0)	2,680 (0.9)	1,331 (1.0)	0.9	3,647 (0.9)	2,345 (0.8)	1,302 (0.9)	0.9	3,959 (0.9)	2,403 (0.9)	1,556 (0.9)	0.6
Normal	3,215 (0.8)	2,197 (0.8)	1,018 (0.8)	0.4	3,124 (0.7)	2,102 (0.7)	1,022 (0.8)	0.6	2,781 (0.7)	1,850 (0.7)	0,931 (0.7)	0.0	2,862 (0.7)	1,743 (0.7)	1,119 (0.6)	0.6
COPD Severity																
Severe	9,879 (2.4)	6,684 (2.3)	3,195 (2.5)	1.2	10,637 (2.5)	7,089 (2.4)	3,548 (2.7)	1.6	9,033 (2.2)	5,826 (2.1)	3,207 (2.3)	1.3	9,941 (2.3)	5,759 (2.3)	4,182 (2.4)	0.8
Unknown	410,328 (97.6)	284,301 (97.7)	126,027 (97.5)	1.2	410,462 (97.5)	282,356 (97.6)	128,106 (97.3)	1.6	407,634 (97.8)	271,291 (97.9)	136,343 (97.7)	1.3	422,671 (97.7)	250,110 (97.7)	172,561 (97.6)	0.8
Pulmonary Rehab	66,822 (15.9)	47,941 (16.5)	18,881 (14.6)	5.1	68,232 (16.2)	48,659 (16.8)	19,573 (14.9)	5.3	67,976 (16.3)	46,981 (17.0)	20,995 (15.0)	5.2	71,431 (16.5)	45,094 (17.6)	26,337 (14.9)	7.4
Oxygen Therapy	8,163 (1.9)	5,799 (2.0)	2,364 (1.8)	1.2	8,867 (2.1)	6,343 (2.2)	2,524 (1.9)	1.9	8,974 (2.2)	6,095 (2.2)	2,879 (2.1)	0.9	9,039 (2.1)	5,632 (2.2)	3,407 (1.9)	1.9
Engagement Score	10.6 (2.5)	10.8 (2.3)	10.0 (2.9)	30.3	10.3 (2.8)	10.6 (2.6)	9.7 (3.1)	29.5	10.2 (2.9)	10.4 (2.7)	9.6 (3.2)	27.7	10.1 (3.0)	10.3 (2.8)	9.7 (3.2)	19.7
Inpatient Visit	0.4 (0.0)	0.4 (0.0)	0.5 (0.0)	0.0	0.4 (0.0)	0.4 (0.0)	0.5 (0.0)	0.0	0.4 (0.0)	0.4 (0.0)	0.5 (0.0)	0.0	0.4 (0.0)	0.4 (0.0)	0.4 (0.0)	0.0
Outpatient Visit	24.2 (13.0)	24.8 (14.0)	22.6 (12.0)	0.0	25.0 (14.0)	25.8 (14.0)	23.3 (12.0)	0.0	26.5 (14.0)	27.4 (15.0)	24.8 (13.0)	0.0	27.9 (15.0)	28.3 (16.0)	27.3 (15.0)	0.0
Inpatient Respiratory Related	0.1 (0.0)	0.1 (0.0)	0.1 (0.0)	0.0	0.1 (0.0)	0.1 (0.0)	0.1 (0.0)	0.0	0.1 (0.0)	0.1 (0.0)	0.1 (0.0)	0.0	0.1 (0.0)	0.1 (0.0)	0.1 (0.0)	0.0
Hospitalization due to Circulatory Dx	0.3 (0.0)	0.3 (0.0)	0.2 (0.0)	0.0	0.2 (0.0)	0.2 (0.0)	0.2 (0.0)	0.0	0.3 (0.0)	0.3 (0.0)	0.2 (0.0)	0.0	0.3 (0.0)	0.3 (0.0)	0.3 (0.0)	0.0
Comorbidity																

Congestive Heart Failure	88,060 (21.0)	61,025 (21.0)	27,035 (20.9)	0.1	89,030 (21.1)	61,287 (21.2)	27,743 (21.1)	0.2	89,082 (21.4)	59,370 (21.4)	29,712 (21.3)	0.3	91,104 (21.1)	54,732 (21.4)	36,372 (20.6)	2.0
Chronic Obstructive Pulmonary Disease	420,207 (100.0)	290,985 (100.0)	129,222 (100.0)	0.0	421,099 (100.0)	289,445 (100.0)	131,654 (100.0)	0.0	416,667 (100.0)	277,117 (100.0)	139,550 (100.0)	0.0	432,612 (100.0)	255,869 (100.0)	176,743 (100.0)	0.0
Cancer	64,614 (15.4)	45,668 (15.7)	18,946 (14.7)	2.9	63,982 (15.2)	44,953 (15.5)	19,029 (14.5)	3.0	64,090 (15.4)	43,819 (15.8)	20,271 (14.5)	3.6	68,051 (15.7)	41,773 (16.3)	26,278 (14.9)	4.0
Metastasis Cancer	5,472 (1.3)	3,122 (1.1)	2,350 (1.8)	6.2	5,622 (1.3)	3,161 (1.1)	2,461 (1.9)	6.4	5,912 (1.4)	3,274 (1.2)	2,638 (1.9)	5.8	6,269 (1.4)	3,124 (1.2)	3,145 (1.8)	4.6
Cardiovascular Disease	47,925 (11.4)	32,961 (11.3)	14,964 (11.6)	0.8	47,832 (11.4)	32,712 (11.3)	15,120 (11.5)	0.6	46,495 (11.2)	30,824 (11.1)	15,671 (11.2)	0.3	48,719 (11.3)	29,162 (11.4)	19,557 (11.1)	1.1
Dementia	12,518 (3.0)	7,237 (2.5)	5,281 (4.1)	9.0	12,154 (2.9)	7,056 (2.4)	5,098 (3.9)	8.2	11,416 (2.7)	6,564 (2.4)	4,852 (3.5)	6.6	11,601 (2.7)	6,063 (2.4)	5,538 (3.1)	4.7
Diabetes Miletus with complication	39,933 (9.5)	29,434 (10.1)	10,499 (8.1)	6.9	41,147 (9.8)	30,073 (10.4)	11,074 (8.4)	6.8	42,811 (10.3)	30,612 (11.0)	12,199 (8.7)	7.7	47,005 (10.9)	30,349 (11.9)	16,656 (9.4)	7.9
Diabetes Miletus without complication	138,875 (33.0)	100,434 (34.5)	38,441 (29.7)	10.2	141,027 (33.5)	101,137 (34.9)	39,890 (30.3)	9.9	140,971 (33.8)	98,259 (35.5)	42,712 (30.6)	10.3	148,012 (34.2)	91,967 (35.9)	56,045 (31.7)	9.0
Human Immunodeficiency Virus	1,415 (0.3)	1,083 (0.4)	0,332 (0.3)	2.1	1,487 (0.4)	1,118 (0.4)	0,369 (0.3)	1.8	1,592 (0.4)	1,148 (0.4)	0,444 (0.3)	1.6	1,649 (0.4)	1,037 (0.4)	0,612 (0.3)	1.0
Hypertension with complication	54,471 (13.0)	37,454 (12.9)	17,017 (13.2)	0.9	55,168 (13.1)	37,648 (13.0)	17,520 (13.3)	0.9	53,921 (12.9)	35,566 (12.8)	18,355 (13.2)	0.9	56,905 (13.2)	33,954 (13.3)	22,951 (13.0)	0.8
Hypertension without complication	287,070 (68.3)	203,480 (69.9)	83,590 (64.7)	11.2	286,072 (67.9)	201,322 (69.6)	84,750 (64.4)	11.0	280,986 (67.4)	191,905 (69.3)	89,081 (63.8)	11.5	291,475 (67.4)	178,248 (69.7)	113,227 (64.1)	11.9
Liver Disease Mild	16,899 (4.0)	10,770 (3.7)	6,129 (4.7)	5.2	18,040 (4.3)	11,548 (4.0)	6,492 (4.9)	4.6	19,002 (4.6)	11,764 (4.2)	7,238 (5.2)	4.4	21,189 (4.9)	11,613 (4.5)	9,576 (5.4)	4.0
Liver Disease Severe	2,207 (0.5)	1,362 (0.5)	0,845 (0.7)	2.5	2,347 (0.6)	1,459 (0.5)	0,888 (0.7)	2.2	2,573 (0.6)	1,559 (0.6)	1,014 (0.7)	2.0	2,749 (0.6)	1,468 (0.6)	1,281 (0.7)	1.9
Myocardial Infarction History	36,243 (8.6)	24,480 (8.4)	11,763 (9.1)	2.4	36,456 (8.7)	24,161 (8.3)	12,295 (9.3)	3.5	35,670 (8.6)	22,824 (8.2)	12,846 (9.2)	3.4	36,939 (8.5)	21,292 (8.3)	15,647 (8.9)	1.9
Paraplegia/Hemiplegia	4,920 (1.2)	3,176 (1.1)	1,744 (1.3)	2.4	4,996 (1.2)	3,208 (1.1)	1,788 (1.4)	2.3	5,048 (1.2)	3,118 (1.1)	1,930 (1.4)	2.3	5,504 (1.3)	3,057 (1.2)	2,447 (1.4)	1.7
Peptic Ulcer Disease	7,140 (1.7)	4,748 (1.6)	2,392 (1.9)	1.7	6,667 (1.6)	4,421 (1.5)	2,246 (1.7)	1.4	6,429 (1.5)	4,099 (1.5)	2,330 (1.7)	1.5	6,500 (1.5)	3,733 (1.5)	2,767 (1.6)	0.9
Peripheral Vascular Disease	71,663 (17.1)	49,866 (17.1)	21,797 (16.9)	0.7	71,303 (16.9)	49,308 (17.0)	21,995 (16.7)	0.9	70,404 (16.9)	47,280 (17.1)	23,124 (16.6)	1.3	74,510 (17.2)	45,180 (17.7)	29,330 (16.6)	2.8
Rheumatic Arthritis	10,478 (2.5)	7,684 (2.6)	2,794 (2.2)	3.1	10,502 (2.5)	7,625 (2.6)	2,877 (2.2)	2.9	10,308 (2.5)	7,236 (2.6)	3,072 (2.2)	2.7	11,007 (2.5)	7,035 (2.7)	3,972 (2.2)	3.2
Renal Disease	67,153 (16.0)	46,952 (16.1)	20,201 (15.6)	1.4	68,911 (16.4)	47,721 (16.5)	21,190 (16.1)	1.1	68,985 (16.6)	46,282 (16.7)	22,703 (16.3)	1.2	73,356 (17.0)	44,608 (17.4)	28,748 (16.3)	3.1
Renal Failure	67,273 (16.0)	47,048 (16.2)	20,225 (15.7)	1.4	69,004 (16.4)	47,769 (16.5)	21,235 (16.1)	1.0	69,077 (16.6)	46,345 (16.7)	22,732 (16.3)	1.2	73,447 (17.0)	44,658 (17.5)	28,789 (16.3)	3.1
Medication																
Aldosterone	1,629 (0.4)	1,135 (0.4)	494 (0.4)	0.1	1,603 (0.4)	1,111 (0.4)	492 (0.4)	0.2	1,601 (0.4)	1,072 (0.4)	529 (0.4)	0.1	1,617 (0.4)	949 (0.4)	668 (0.4)	0.1
Antibiotic	201,162 (47.9)	137,557 (47.3)	63,605 (49.2)	3.9	207,771 (49.3)	142,068 (49.1)	65,703 (49.9)	1.6	203,190 (48.8)	134,674 (48.6)	68,516 (49.1)	1.0	216,990 (50.2)	127,829 (50.0)	89,161 (50.4)	1.0
Anticholinergic	137,067 (32.6)	97,531 (33.5)	39,536 (30.6)	6.3	149,953 (35.6)	105,995 (36.6)	43,958 (33.4)	6.8	155,978 (37.4)	106,969 (38.6)	49,009 (35.1)	7.2	163,129 (37.7)	99,018 (38.7)	64,111 (36.3)	5.0
Antidepressant	157,300 (37.4)	109,179 (37.5)	48,121 (37.2)	0.6	159,080 (37.8)	110,141 (38.1)	48,939 (37.2)	1.8	159,355 (38.2)	106,952 (38.6)	52,403 (37.6)	2.1	167,661 (38.8)	99,426 (38.9)	68,235 (38.6)	0.5
Antiepileptic	90,505 (21.5)	63,506 (21.8)	26,999 (20.9)	2.3	96,316 (22.9)	67,121 (23.2)	29,195 (22.2)	2.4	102,267 (24.5)	68,926 (24.9)	33,341 (23.9)	2.3	112,791 (26.1)	66,979 (26.2)	45,812 (25.9)	0.6
Antipsychotic	38,506 (9.2)	25,132 (8.6)	13,374 (10.3)	5.8	37,737 (9.0)	24,523 (8.5)	13,214 (10.0)	5.4	37,017 (8.9)	23,468 (8.5)	13,549 (9.7)	4.3	37,757 (8.7)	20,918 (8.2)	16,839 (9.5)	4.8
Antithrombotic	137,263 (32.7)	95,439 (32.8)	41,824 (32.4)	0.9	139,688 (33.2)	96,658 (33.4)	43,030 (32.7)	1.5	140,387 (33.7)	94,170 (34.0)	46,217 (33.1)	1.8	148,391 (34.3)	88,251 (34.5)	60,140 (34.0)	1.0
Aspirin	107,103 (25.5)	74,683 (25.7)	32,420 (25.1)	1.3	107,889 (25.6)	75,036 (25.9)	32,853 (25.0)	2.2	106,032 (25.4)	71,271 (25.7)	34,761 (24.9)	1.9	109,362 (25.3)	64,881 (25.4)	44,481 (25.2)	0.4
Beta 2 Agonist	228,820 (54.5)	161,331 (55.4)	67,489 (52.2)	6.5	231,187 (54.9)	161,474 (55.8)	69,713 (53.0)	5.7	237,372 (57.0)	159,973 (57.7)	77,399 (55.5)	4.6	252,143 (58.3)	149,803 (58.5)	102,340 (57.9)	1.3
Beta Blocker	186,606 (44.4)	131,532 (45.2)	55,074 (42.6)	5.2	189,999 (45.1)	133,206 (46.0)	56,793 (43.1)	5.8	189,402 (45.5)	128,632 (46.4)	60,770 (43.5)	5.8	196,434 (45.4)	118,767 (46.4)	77,667 (43.9)	5.0
Bronchodilator	294,937 (70.2)	207,825 (71.4)	87,112 (67.4)	8.7	300,256 (71.3)	210,167 (72.6)	90,089 (68.4)	9.2	299,613 (71.9)	202,896 (73.2)	96,717 (69.3)	8.6	313,998 (72.6)	187,851 (73.4)	126,147 (71.4)	4.6
Calcium Channel Blocker	121,948 (29.0)	85,896 (29.5)	36,052 (27.9)	3.6	122,289 (29.0)	85,983 (29.7)	36,306 (27.6)	4.7	120,641 (29.0)	82,149 (29.6)	38,492 (27.6)	4.6	125,520 (29.0)	75,760 (29.6)	49,760 (28.2)	3.2
Clopidogrel	39,871 (9.5)	28,496 (9.8)	11,375 (8.8)	3.4	41,314 (9.8)	29,420 (10.2)	11,894 (9.0)	3.8	40,795 (9.8)	28,201 (10.2)	12,594 (9.0)	3.9	42,275 (9.8)	25,822 (10.1)	16,453 (9.3)	2.6

Digoxin	22,401 (5.3)	15,664 (5.4)	6,737 (5.2)	0.8	20,939 (5.0)	14,614 (5.0)	6,325 (4.8)	1.1	19,786 (4.7)	13,435 (4.8)	6,351 (4.6)	1.4	18,501 (4.3)	11,283 (4.4)	7,218 (4.1)	1.6
Diuretics	173,679 (41.3)	122,262 (42.0)	51,417 (39.8)	4.5	172,808 (41.0)	121,197 (41.9)	51,611 (39.2)	5.4	168,777 (40.5)	114,726 (41.4)	54,051 (38.7)	5.4	172,304 (39.8)	104,511 (40.8)	67,793 (38.4)	5.1
Methylxanthines	9,197 (2.2)	6,886 (2.4)	2,311 (1.8)	4.1	8,124 (1.9)	6,135 (2.1)	1,989 (1.5)	4.6	7,277 (1.7)	5,288 (1.9)	1,989 (1.4)	3.8	6,742 (1.6)	4,340 (1.7)	2,402 (1.4)	2.7
Mucolytics	4,332 (1.0)	2,994 (1.0)	1,338 (1.0)	0.1	3,005 (0.7)	2,081 (0.7)	0,924 (0.7)	0.2	2,424 (0.6)	1,620 (0.6)	0,804 (0.6)	0.1	2,654 (0.6)	1,592 (0.6)	1,062 (0.6)	0.3
Opiate	191,255 (45.5)	130,712 (44.9)	60,543 (46.9)	3.9	194,464 (46.2)	132,162 (45.7)	62,302 (47.3)	3.3	192,598 (46.2)	126,813 (45.8)	65,785 (47.1)	2.8	197,486 (45.6)	114,836 (44.9)	82,650 (46.8)	3.8
Protein pump inhibitor	199,578 (47.5)	142,285 (48.9)	57,293 (44.3)	9.2	201,729 (47.9)	142,965 (49.4)	58,764 (44.6)	9.5	200,469 (48.1)	137,853 (49.7)	62,616 (44.9)	9.8	207,568 (48.0)	126,853 (49.6)	80,715 (45.7)	7.8
Renin Angiotensin Inhibitor	187,419 (44.6)	133,106 (45.7)	54,313 (42.0)	7.5	187,781 (44.6)	132,425 (45.8)	55,356 (42.0)	7.5	185,242 (44.5)	126,528 (45.7)	58,714 (42.1)	7.2	191,115 (44.2)	116,168 (45.4)	74,947 (42.4)	6.0
Spironolactone	16,777 (4.0)	11,868 (4.1)	4,909 (3.8)	1.4	17,186 (4.1)	12,148 (4.2)	5,038 (3.8)	1.9	17,611 (4.2)	11,962 (4.3)	5,649 (4.0)	1.3	18,221 (4.2)	10,982 (4.3)	7,239 (4.1)	1.0
Statin	263,210 (62.6)	190,111 (65.3)	73,099 (56.6)	18.0	262,738 (62.4)	188,932 (65.3)	73,806 (56.1)	18.9	260,534 (62.5)	181,631 (65.5)	78,903 (56.5)	18.5	271,642 (62.8)	168,541 (65.9)	103,101 (58.3)	15.6
Steroid	111,634 (26.6)	76,813 (26.4)	34,821 (26.9)	1.2	118,542 (28.2)	81,446 (28.1)	37,096 (28.2)	0.1	119,176 (28.6)	79,403 (28.7)	39,773 (28.5)	0.3	130,801 (30.2)	77,374 (30.2)	53,427 (30.2)	0.0
